# The impact of dementia on rehabilitation outcomes following hip fracture

**DOI:** 10.1002/agm2.12251

**Published:** 2023-04-20

**Authors:** Yee Leng Loh, John Wicks, Tara Alexander

**Affiliations:** ^1^ Complex Management Unit Gold Coast University Hospital Southport Queensland Australia; ^2^ Department of Rehabilitation, Robina Hospital Robina Queensland Australia; ^3^ Australasian Rehabilitation Outcome Centre, Australian Health Services Research Institute University of Wollongong Australia North Wollongong New South Wales Australia

**Keywords:** dementia, hip fracture, rehabilitation

## Abstract

**Objective:**

To compare clinical outcomes between patients for whom their participation in inpatient rehabilitation was and was not impacted by dementia through matching patients reporting dementia (dementia group) with those not reporting dementia (non‐dementia group).

**Methods:**

Prospectively collected data held by the Australasian Rehabilitation Outcome Centre (AROC) were analyzed for patients aged 65 years or older receiving inpatient rehabilitation in public hospitals in Australia following a hip fracture and discharged between July 1, 2014, and June 30, 2019. Patients reported as having dementia impacting their rehabilitation program were matched to patients not reporting dementia based on age, admission motor Functional Independence Measure (FIM) score, and accommodation prior to rehabilitation. The matched cohorts were compared in relation to clinical outcomes (motor and cognitive FIM improvement, FIM efficiency, length of stay, and discharge destination) following participation in hospital‐based rehabilitation using univariate analysis.

**Results:**

Patients with dementia had significantly lower cognitive FIM scores on commencing rehabilitation (17.6 and 26.9, respectively, *P* < 0.001) and their median length of stay was 2 days shorter than those without dementia (21 and 23 days, respectively, *P* < 0.001). Relative change in FIM score and FIM efficiency (per week) were lower in the dementia group [relative FIM score change of dementia vs non‐dementia, respectively, 26.2% *vs*. 44.0% (*P* < 0.001) and FIM efficiency, 6.5 *vs*. 8.9 (*P* < 0.001)]. Discharge destination between the two groups was statistically different, with 35.7% of patients with dementia being discharged to residential aged care facilities (RACFs) compared to 21.7% of those without dementia (*P* < 0.001). More patients with dementia had carers in their private residence in the post‐rehabilitation phase, 82.2% *vs*. 57.6% (*P* < 0.001).

**Conclusion:**

Patients with dementia who sustain a fractured hip benefit from inpatient rehabilitation, although their clinical outcomes are not as good as those without dementia. FIM change and FIM efficiency were lower in the dementia group. Length of stay in the hospital for patients with dementia was shorter due to earlier recognition for the need for placement in either an RACF or at home with carer support. The need for placement in an RACF or carer support in a private residence was significantly greater in the dementia group.

## Graphical abstract

Patients with dementia who sustain a fractured hip benefit from inpatient rehabilitation though their clinical outcomes are not as good as those without dementia. FIM change and FIM efficiency were lower in the dementia group. Length of stay in hospital for dementia patients was shorter due to earlier recognition for the need for placement in either a RACF or at home with carer support.

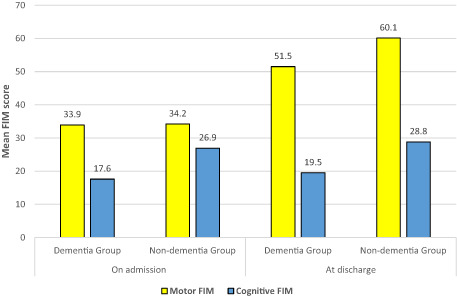

## 1 INTRODUCTION

Dementia describes a syndrome characterized by gradual impairment of brain functions. It affects memory, thinking, orientation, comprehension, calculation, learning capacity, language, and judgment.^1^ Worldwide, around 50 million people have dementia and almost 10 million new cases are diagnosed yearly. The total number of people with dementia is projected to reach 82 million in 2030 and 152 million in 2050.^1^, ^2^ In Australia, an estimated 472,000 Australians had dementia in 2021.^3^ In 2020, dementia was estimated to cost Australia more than $15 billion, with a cost estimate of $18.7 billion in 2023, increasing to $36.8 billion by 2056.^4^

Hip fractures contribute to both mortality and morbidity in the elderly. Dementia is associated with a hip fracture hospitalization rate 2.5 times that of adults without dementia.^5^ In Australia, between 2015 and 2016, there were 50,900 episodes of hip fractures that required hospital care for both new hip fractures and management and repair of previous fractures. These hospitalizations accounted for 579,000 bed days and required 206,300 procedures or interventions.^6^ A meta‐analysis revealed that women sustaining a hip fracture had a fivefold increase and men almost an eightfold increase in relative likelihood of death within the first 3 months as compared with age‐ and sex‐matched controls.^7^ The CHANCES project, a follow‐up of 122,808 participants from eight cohorts in Europe and the United States for a mean of 12.6 years reported 4273 incident hip fractures and 27,999 deaths, with both increased short‐ and long‐term all‐cause mortality for both sexes.^8^

The changing demographic of dementia worldwide will continue to impact on hip fracture rehabilitation. Fragility fractures are relatively common in the elderly, particularly among those with dementia. Studies exploring the predictors of poor clinical outcomes and level of function on discharge for those who underwent rehabilitation in subacute settings are limited. A recent Cochrane review of hip fracture management by Smith et al suggested that models of enhanced rehabilitation and care used in the included trials show benefits over usual care for preventing delirium and reducing the length of stay (LOS) for people with dementia. However, the level of evidence of these results is low as data were available from only seven trials with a total of 555 participants.^9^ This highlights the importance of further research to define optimal rehabilitation and supportive care for people with dementia following hip fracture surgery.

## 2 AIM OF THE STUDY

There were two objectives of this study. First, we described the patient profiles on admission to inpatient rehabilitation for those who did and did not report dementia impacting their rehabilitation. Second, we compared patient profiles and clinical outcomes for patients who reported dementia impacting on their participation in rehabilitation (dementia group) to a matched cohort of those who did not report dementia (non‐dementia group).

## 3 METHODS

### 3.1 Study design

This study used prospectively collected episodes of inpatient rehabilitation routinely submitted to the Australasian Rehabilitation Outcomes Centre (AROC) for benchmarking. The AROC is the national rehabilitation medicine integrated outcomes center of Australia and New Zealand. It is a joint initiative of the Australian rehabilitation sector (providers, funders, regulators, and consumers)^10^, ^11^ and collects data on over 95% of inpatient rehabilitation services across both countries as members.

## 4 STUDY POPULATION

Patients receiving inpatient rehabilitation following a fractured neck of femur, who were aged 65 years or older and were treated in an Australian public hospital between July 1, 2014, and June 30, 2019, were included in this study.

### 4.1 Identifying patients with dementia

The AROC dataset includes comorbidities, defined as pre‐existing illness/impairments, not part of the principal presenting condition, impacting the rehabilitation process. Dementia is one such comorbidity.

### 4.2 Data collection

The AROC data collection includes:

- On admission only: age, sex, and date of fracture.
- On admission and discharge: accommodation, carer status, services received, and functional status.

### 4.3 Clinical outcome measures

#### 4.3.1 Length of stay

An inpatient rehabilitation episode of care is the date that the patient’s care is transferred to a rehabilitation physician or physician with interest in rehabilitation, and it is recorded in the medical record that the rehabilitation team has commenced the rehabilitation program/provision of care.^12^ Episode end date records the date that the patient completes their rehabilitation episode; it defines the end of rehabilitation episode and is the date of which the LOS concludes. An inpatient rehabilitation episode of care ends when the patient is discharged from the rehabilitation unit and/or the care type is changed from rehabilitation to acute or some other subacute (maintenance/palliative care).

#### 4.3.2 Functional improvement

The Functional Independence Measure (FIM) instrument is a primary indicator of patient disability with FIM being used to track changes in functional ability of a patient during an episode of hospital rehabilitation care.^11^ Each patient’s level of function is assessed using the FIM instrument at the start of a rehabilitation episode of care and again at the end of a rehabilitation episode of care. FIM is comprised of 18 items (13 motor and 5 cognitive); each item is scored on a 7‐point ordinal scale (1 = complete dependence to 7 = complete independence). The total score for the FIM is a value between 18 and 126. FIM change is defined as a difference in the FIM score between admission and discharge. FIM efficiency is defined by the amount of FIM change per day in inpatient rehabilitation (FIM change / LOS).

### 4.4 Statistical Analysis

To create the matched cohort, patients with dementia (dementia group) were matched by age, motor functional status on admission, and residence at the time of fracture with patients not reporting dementia (non‐dementia group). Four passes were required to obtain the full matched cohort (Table 1), with subsequent passes being less rigid. Most dementia episodes (96%) found a match in the first pass, giving a match that was within 1 year of age and one point of motor admission FIM score and came from the same prior accommodation.

**TABLE 1:** Matching details

Criteria for match				Matches	Total	Cumulative
Pass	Age	Admission FIM motor	Accommodation			
1	+/− 1 y	+/− 1 point	Same	1829	96.2%	96.2%
2	+/− 2 y	+/− 2 points	Same	36	1.9%	98.1%
3	+/− 3 y	+/− 3 points	Same	8	0.4%	98.5%
4	+/− 1 y	+/− 1 point	Any	28	1.5%	100.0%

FIM, Functional Independence Measure.

Univariate analyses were used to describe group differences; the chi‐square test was used with categorical variables, and the *t* test was used with continuous variables. A *P* value < 0.05 was regarded as statistically significant.

The Australian National Subacute and Non Acute Patient (AN‐SNAP) is a case‐mix classification that categorizes rehabilitation, palliative care, geriatric evaluation and management, psychogeriatric care, and non‐acute care. The rehabilitation branch classifies patients based on impairment, level of function on admission, and age.^13^, ^14^ In Australia, AN‐SNAP is used for funding and case‐mix adjustment of clinical outcomes. Version 4 AN‐SNAP was used in this study.

## 5 RESULTS

### 5.1 Complete Cohort

During the study period 20,905 episodes of care were admitted to inpatient rehabilitation units following a fractured neck of femur. Of these, 1901 episodes reported dementia impacted participation in rehabilitation. Table 2 describes the patient profiles on admission to inpatient rehabilitation for those who did and did not report dementia impacting their rehabilitation.

**TABLE 2:** Patient profiles on admission to inpatient rehabilitation for those who did and did not report dementia impacting their rehabilitation using the complete cohort

Comparison of admission profile of patients	Reported dementia impacting rehabilitation?			
	Yes		No	
	n	%	n	%
All episodes	1901		19,004	
Age group				
65–69 y	34	1.8%	1455	7.7%
70–74 y	105	5.5%	2235	11.8%
75–79 y	232	12.2%	3047	16.0%
80–84 y	450	23.7%	4418	23.2%
85–89 y	565	29.7%	4527	23.8%
90–94 y	403	21.2%	2579	13.6%
95+ y	112	5.9%	743	3.9%
Mean (95% CI)	85.2	(84.9–85.5)	82.0	(81.9–82.1)
Median (IQR)	86	(81–90)	83	(77–88)
Sex				
Male	585	30.8%	5849	30.8%
Female	1316	69.2%	13,154	69.2%
Accommodation prior to rehabilitation				
Private residence	1530	80.5%	17,139	90.2%
RACF	325	17.1%	1021	5.4%
Other	46	2.4%	844	4.4%
Carer prior to rehabilitation (private residence)				
Has carer	1086	71.0%	6857	40.0%
Needs carer	120	7.8%	744	4.3%
Does not need carer	312	20.4%	9280	54.1%
Any services received prior to rehabilitation? (Private residence)				
Yes	671	43.9%	5871	34.3%
If yes, what type of services were received?				
Domestic assistance	538	80.2%	4938	84.1%
Social support	189	28.2%	944	16.1%
Nursing care	59	8.8%	426	7.3%
Allied health care	23	3.4%	210	3.6%
Personal care	271	40.4%	1264	21.5%
Meals	214	31.9%	1152	19.6%
Provision of goods and equipment	63	9.4%	507	8.6%
Transport services	175	26.1%	1016	17.3%
Case management	39	5.8%	360	6.1%
Time since hip fracture				
Within a wk	693	36.5%	7765	40.9%
Within 2 wk	586	30.8%	5542	29.2%
Within a month	392	20.6%	3295	17.3%
Within 3 month	121	6.4%	1040	5.5%
Within 6 month	9	0.5%	109	0.6%
Greater than 6 month	7	0.4%	52	0.3%
AN‐SNAP class				
4AH1 (orthopedic conditions, fractures, weighted FIM motor 49–91, and FIM cognition 33–35)	35	1.8%	4324	22.8%
4AH2 (orthopedic conditions, fractures, weighted FIM motor 49–91, and FIM cognition 5–32)	396	20.8%	4930	25.9%
4AH3 (orthopedic conditions, fractures, and weighted FIM motor 38–48)	355	18.7%	4077	21.5%
4AH4 (orthopedic conditions, fractures, and weighted FIM motor 19–37)	746	39.2%	4955	26.1%
4AZ3 (weighted FIM motor score 13–18, all other impairments, age ≥ 65 y)	369	19.4%	698	3.7%
Motor FIM admission score				
Mean (95% CI)	33.9	(33.2–34.6)	46.0	(45.7–46.2)
Median (IQR)	32	(21–46)	47	(35–57)
Cognition FIM admission score				
Mean (95% CI)	17.6	17.2–17.9	28.0	(28.0–28.6)
Median (IQR)	18	11–23	30	(25–34)

AN‐SNAP, Australian National Subacute and Non Acute Patient; CI, confidence interval; FIM, Functional Independence Measure; IQR, interquartile range; RACF, residential aged care facility.

Most patients were admitted to the rehabilitation unit within 2 weeks since the initial injury with 67.3% in the dementia group (n = 1279/1901) *vs*. 70.1% in the non‐dementia group (n = 13,307/19,004). Most of the admitted patients were aged between 80 and 94 years, accounting for 74.6% and 60.6%, respectively, in the dementia and non‐dementia groups. Both groups had a similar proportion of extremely old people aged 95 years or older (5.9% in the dementia group and 3.9% in the non‐dementia group). Of the 20,905 patients in this study, 69.2% were women in both groups. A large proportion of the patients were from private residences at the time of admission, 80.5% in the dementia group and 90.2% in the non‐dementia group. Compared to those without dementia, patients with dementia admitted for hip fracture rehabilitation had a higher likelihood of residing in residential aged care facilities (RACFs; 17.1% in the dementia group vs 5.4% in the non‐dementia group). At the time of admission, 71% of patients with dementia had carer assistance compared with 40% for non‐dementia patients. Service requirements were similar between the dementia and non‐dementia groups for domestic assistance (80.2% *vs*. 84.1%, respectively), with increased requirements for both personal care (40.4% *vs*. 21.5%, respectively) and meal preparation (31.9% *vs*. 19.6%, respectively) in the dementia group.

Patients with dementia had lower motor FIM scores (mean 33.9 and median 32) on admission compared to those without dementia (mean 46.0 and median 47). Similarly, patients with dementia had significantly lower cognitive FIM scores (mean 17.6 and median 18) than those without dementia (mean 28.0 and median 30).

### 5.2 Matched Cohort

After controlling for age, motor function, and prior accommodation by matching, there was no difference between the two groups in the timing of the rehabilitation unit admission since the initial insult (*P* = 0.074; Table 3). Admissions from a private residence showed both higher needs for carer assistance and support services (71.0% and 43.9%, respectively) for the dementia cohort compared with the non‐dementia cohort (47.8% and 37.1%, respectively). Further, mean admission cognitive FIM was significantly lower in the dementia group compared with the non‐dementia group (17.6 and 26.9 points, respectively, *P* < 0.001).

**TABLE 3:** Patient profiles on admission to inpatient rehabilitation for those who did and did not report dementia impacting their rehabilitation using the matched cohort

	Reported dementia impacting rehabilitation?			
	Yes		Matched no	
	n	%	n	%
All episodes	1901		1901	
Age group (chi‐square χ^2^ *=* 1.867, *df* = 6, *P* = 0.92; *t* test t = −0.150, *df* = 3800, *P* = 0.880)				
65–69 y	34	1.8%	41	2.2%
70–74 y	105	5.5%	92	4.8%
75–79 y	232	12.2%	224	11.8%
80–84 y	450	23.7%	460	24.2%
85–89 y	565	29.7%	570	30.0%
90–94 y	403	21.2%	406	21.4%
95+ y	112	5.9%	108	5.7%
Mean (95% CI)	85.2	(84.9–85.5)	85.1	(84.8–85.4)
Median (IQR)	86	(81–90)	86	(81–90)
Sex (chi‐square χ^2^ = 0.150, *df* = 1, *P* = 0.698)				
Male	585	30.8%	574	30.2%
Female	1316	69.2%	1327	69.8%
Accommodation prior to rehabilitation (chi‐square χ^2^ = 2.346, *df* = 2, *P* = 0.309)				
Private residence	1530	80.5%	1548	81.4%
RACF	325	17.1%	297	15.6%
Other	46	2.4%	56	2.9%
Carer prior to rehabilitation (private residence) (chi‐square χ^2^ = 665.287, *df* = 4, *P* < 0.001)				
Has carer	1086	71.0%	740	47.8%
Needs carer	120	7.8%	94	6.1%
Does not need carer	312	20.4%	688	44.4%
Any services received prior to rehabilitation? (private residence) (chi‐square χ^2^ = 14.388, *df* = 2, *P* = 0.001)				
Yes	671	43.9%	575	37.1%
If yes, what type of services were received? (those with * are statistically different)				
Domestic assistance	538	80.2%	476	82.8%
*Social support	189	28.2%	90	15.7%
Nursing care	59	8.8%	57	9.9%
Allied health care	23	3.4%	32	5.6%
*Personal care	271	40.4%	186	32.3%
*Meals	214	31.9%	118	20.5%
Provision of goods and equipment	63	9.4%	46	8.0%
*Transport services	175	26.1%	107	18.6%
Case management	39	5.8%	33	5.7%
Time since hip fracture (chi‐square χ^2^ = 14.303, *df* = 8, *P* = 0.074)				
Within a week	693	36.5%	663	34.9%
Within 2 wk	586	30.8%	569	29.9%
Within a month	392	20.6%	368	19.4%
Within 3 mo	121	6.4%	160	8.4%
Within 6 mo	9	0.5%	21	1.1%
Greater than 6 mo	7	0.4%	7	0.4%
AN‐SNAP class (chi‐square χ^2^ = 665.287, *df* = 2, *P* < 0.001)				
4AH1 (weighted FIM motor score49–91, FIM cognition score 33–35)	35	1.8%	465	24.5%
4AH2 (weighted FIM motor score49–91, FIM cognition score5–32)	396	20.8%	40	2.1%
4AH3 (weighted FIM motor score 38–48)	355	18.7%	370	19.5%
4AH4 (weighted FIM motor score 19–37)	746	39.2%	705	37.1%
4AZ3 (weighted FIM motor score 13–18, age ≥ 65 y)	369	19.4%	321	16.9%
Motor FIM admission score (*t* ‐test t = 0.638, *df* = 3800, *P* = 0.524)				
Mean (95% CI)	33.9	(33.2–34.6)	34.2	(33.6–34.9)
Median (IQR)	32	(21–46)	33	(21–46)
Cognition FIM admission score (*t* test t = 37.027, *df* = 3800, *P* < 0.001)				
Mean (95% CI)	17.6	17.2–17.9	26.9	(26.5–27.2)
Median (IQR)	18	11–23	29	(22–34)

AN‐SNAP, Australian National Subacute and Non Acute Patient; CI, confidence interval; FIM, Functional Independence Measure; IQR, interquartile range; RACF, residential aged care facility.

**FIGURE 1:**
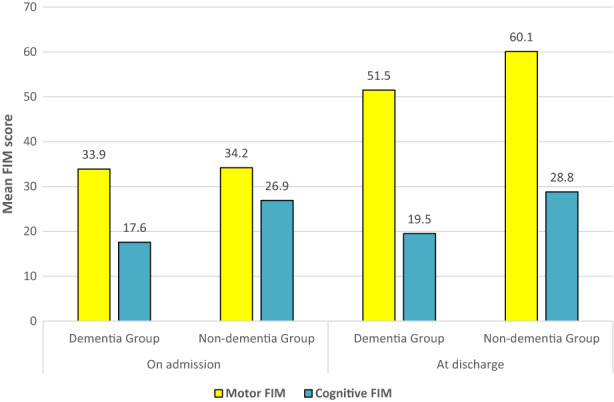
Function outcomes (FIM) at admission to and discharge from inpatient rehabilitation among a matched cohort of patients who did and did not report dementia impacting on their participation. FIM, Functional Independence Measure.

In comparing clinical outcomes (Table 4), this study demonstrated those reporting dementia achieved 8 points less motor FIM improvement from rehabilitation than the matched non‐dementia group (17.6 and 25.9, respectively; Figure 1). By comparison, mean cognitive FIM scores showed an equivalent small increase during rehabilitation (1.9 in both groups). The highest functioning AN‐SNAP classes (4AH1‐3) account for 45% of dementia episodes and have similar motor FIM discharge scores to the non‐dementia group compared to the lower functioning classes. As expected from these results, FIM gain and FIM efficiency were both significantly greater in the non‐dementia cohort (*P* < 0.001).

**TABLE 4:** Comparison of clinical outcomes among patients who did and did not report dementia impacting on their participation in rehabilitation using a matched cohort

	Reported dementia impacting rehabilitation?			
	Yes		Matched no	
	n	%	n	%
All episodes	1901		1901	
Completed rehabilitation program	1538	80.9%	1404	73.9%
	No.	Mean (95% CI)	No.	Mean (95% CI)
Motor FIM discharge score (*t* test t = 12.135, *df* = 2940, *P* < 0.001) (classes with * are statistically different)				
*4AH1 (weighted FIM motor score 49–91, FIM cognition score 33–35)	30	71.5 (67.2–75.9)	405	75.6 (74.8–76.4)
*4AH2 (weighted FIM motor score 49–91, FIM cognition score 5–32)	354	68.8 (67.8–69.9)	33	68.5 (64.2–72.7)
*4AH3 (weighted FIM motor score 38–48)	307	60.0 (58.7–61.3)	274	63.5 (61.9–65.1)
*4AH4 (weighted FIM motor score 19–37)	594	45.9 (44.7–47.1)	496	54.2 (52.7–55.7)
*4AZ3 (weighted FIM motor score 13–18, age ≥ 65 y)	253	27.4 (25.7–29.1)	196	36.7 (34.0–39.5)
All AN‐SNAP classes	1538	51.5 (50.5–52.4)	1404	60.1 (59.1–61.1)
Median (IQR)		54 (38–66)		64.5 (47–75)
Cognition FIM discharge score (*t* test t = 35.856, *df* = 2939, *P* < 0.001)				
*4AH1 (weighted FIM motor score 49–91, FIM cognition score 33–35)	30	32.3 (30.5–34.1)	405	34.5 (34.3–34.6)
*4AH2 (weighted FIM motor score 49–91, FIM cognition score 5–32)	354	23.6 (23.0–24.2)	33	29.1 (27.9–30.3)
*4AH3 (weighted FIM motor score 38–48)	307	21.4 (20.8–22.1)	274	28.5 (27.9–29.1)
*4AH4 (weighted FIM motor score 19–37)	594	18.2 (17.7–18.8)	496	27.5 (27.0–28.0)
*4AZ3 (weighted FIM motor score 13–18, age ≥ 65 y)	253	12.9 (12.1–13.6)	196	20.7 (19.6–21.8)
All AN‐SNAP classes	1538	19.5 (19.1–19.9)	1404	28.8 (28.5–29.1)
Median (IQR)		20 (14–25)		30 (25–35)
Case‐mix adjusted change in FIM score (*t* test t = 15.989, *df* = 2832, *P* < 0.001)				
Mean (95% CI)	1538	−9.4 (−10.1–8.7)	1404	−0.5 (−1.3–0.3)
Median (IQR)		−9.3 (−19.4–0.1)		0.6 (−10.5–9.9)
Relative change in FIM score (*t* test t = 20.386, *df* = 2742, *P* < 0.001)				
Mean (95% CI)	1538	26.2% (25.1%–27.3%)	1404	44.0% (42.7%–45.3%)
Median (IQR)		24.7% (10.1%–41.8%)		47.4% (23.3%–64.3%)
Length of stay in rehabilitation (*t* test t = 3.742, *df* = 2767, *P* < 0.001)				
Mean (95% CI)	1538	23.6 (22.9–24.3)	1404	25.6 (24.8–26.4)
Median (IQR)		21 (15–29)		23 (15–33)
Case‐mix adjusted length of stay in rehabilitation (*t* test t = 6.112, *df* = 2940, *P* < 0.001)				
Mean (95% CI)	1538	−1.9 (−2.6–1.2)	1404	1.4 (0.6–2.2)
Median (IQR)		−3.3 (−10.8–5.2)		−0.6 (−7.6–8.2)
FIM efficiency (per week) (*t* test t = 8.732, *df* = 2814, *P* < 0.001)				
Mean (95% CI)	1538	6.5 (6.1–6.8)	1404	8.9 (8.5–9.3)
Median (IQR)		5.1 (2.4–9.0)		7.5 (4.1–11.6)
Accommodation post rehabilitation (chi‐square χ^2^ = 69.880, *df* = 3, *P* < 0.001)				
Private residence	729	51.8%	885	65.1%
RACF	502	35.7%	295	21.7%
Other	39	2.8%	51	3.8%
Unknown	136	9.7%	129	9.5%
Carer post rehabilitation (private residence) (chi‐square χ^2^ = 132.267, *df* = 5, *P* < 0.001)				
Has carer	599	82.2%	510	57.6%
Needs carer	22	3.0%	35	4.0%
Does not need carer	93	12.8%	318	35.9%
Unknown	15	2.1%	22	2.5%
Any services to be received post rehabilitation? (private residence) (chi‐square χ^2^ = 6.193, *df* = 2, *P* = 0.045)				
Yes	537	73.7%	604	68.2%
If yes, what type of services will be received? (those with * are statistically different)				
Domestic assistance	342	63.7%	404	66.9%
*Social support	164	30.5%	116	19.2%
Nursing care	123	22.9%	159	26.3%
*Allied health care	232	43.2%	349	57.8%
*Personal care	338	62.9%	311	51.5%
*Meals	125	23.3%	102	16.9%
*Provision of goods and equipment	185	34.5%	144	23.8%
Transport services	129	24.0%	157	26.0%
*Case management	115	21.4%	98	16.2%

*Note:* Classes with * are statistically different.

AN‐SNAP, Australian National Subacute and Non Acute Patient; CI, confidence interval; FIM, Functional Independence Measure; IQR, interquartile range; RACF, residential aged care facility.

**FIGURE 2:**
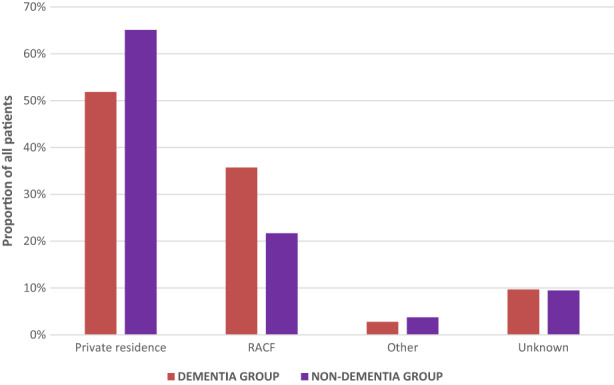
Accommodation at discharge from inpatient rehabilitation among a matched cohort of patients who did and did not report dementia impacting on their participation. RACF, residential care facilities.

**FIGURE 3:**
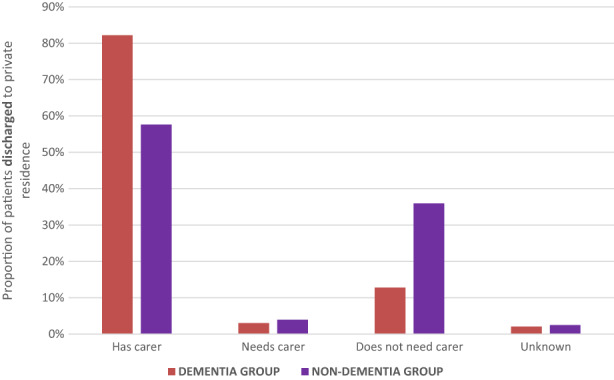
Carer status among patients discharge to a private residence after inpatient rehabilitation among a matched cohort of patients who did and did not report dementia impacting on their participation.

Patients with dementia on admission stayed 2 days less in the rehabilitation ward compared to those without dementia (21 and 23 days, respectively, *P* < 0.001). Patients reporting dementia were statistically more likely to be discharged to an RACF (35.7% and 21.7%, respectively, *P* < 0.001; Figure 2). Patients with dementia were more likely to require carer support in their private residence in the post‐rehabilitation phase (82.2% and 57.6%, respectively, *P* < 0.001; Figure 3).

Both groups showed increased needs for services post‐rehabilitation of almost 30% compared to admission (73.3% and 68.2%, respectively, *P* = 0.045). Allied health care and social support increased in both groups, being greater for the non‐dementia group. Personal care support increased about 20% in both groups, whereas support for meals declined. A higher proportion of patients with dementia (5.8% on admission and 21.4% on discharge) needed case management support in the post‐rehabilitation phase than those without dementia (5.7% on admission and 16.2% on discharge).

## 6 DISCUSSION

Dementia is a common comorbidity in older people admitted to acute hospitals and is associated with poor outcomes.^15^ This is consistent with the patients' profiles of 80–94 years age group in this study.

We demonstrated that both the dementia and matched non‐dementia groups showed improvements in FIM scores. The non‐dementia group showed a greater relative change in FIM score compared to the dementia group, which translated into greater FIM efficiency despite the longer LOS. This is consistent with a study by Heruti et al that found that higher cognitive status at admission was related to higher motor FIM scores at discharge. This study observed lower motor FIM scores in cognitively impaired patients, the relative functional gain in patients with the lowest Mini‐Mental State Examination (MMSE) scores being significantly lower.^16^ Rolland et al showed similar results with patients without cognitive impairment having significantly higher admission and discharge FIM.^17^ A systematic review by Allen et al of patients with dementia following hip fracture reported that people with mild or moderate dementia can show improved function and ambulation and decreased fall risk, similar to gains achieved by those without dementia.^18^ Similar results have been reported by both McGilton et al and Muir et al in using FIM as a measure of mobility in relation to cognitive status with inpatient rehabilitation.^19^, ^20^ Goldstein et al, in examining motor FIM scores in patients with dementia following hip fracture, observed that cognitively impaired patients displayed gains in specific FIM areas (self‐care, sphincter control, and locomotion) similar to cognitively intact patients, with mobility gains being significantly greater in cognitively intact patients.^21^ Lenze et al observed that patients with depression, apathy, or cognitive impairment who received inpatient rehabilitation had significantly better functional outcomes than similarly impaired patients at skilled nursing facilities.^22^

We noted that patients who had dementia on admission had a shorter inpatient rehabilitation stay in the hospital; the median LOS was 2 days less compared to those without dementia (*P* < 0.001) in the matched cohort, as they are more likely to go to an RACF or home with a carer support. This is consistent with recent study by Rasu et al that demonstrated that patients with dementia and without a known diagnosis of osteoporosis had a 5% shorter LOS (*P* = 0.04) when hospitalized for hip fracture.^23^

Discharge destination is an important rehabilitation outcome. This study looked at the accommodation, assistance from a carer, and types of service prior to rehabilitation episodes following inpatient rehabilitation admission. Differences were found among those coming from a private residence with respect to their carer needs and services required, particularly social support, personal care, meals, and transport services. The availability of community support services impacts on dementia management at all stages of this disease. Huusko et al reported the outcome of 243 people aged ≥ 65 years following hip fracture with 91% of patients with mild and 63% of patients with moderate dementia living independently 3 months after postoperative intensive geriatric rehabilitation.^24^

Jorissen et al demonstrated that dementia was associated with a higher risk of 2‐year mortality [hazard ratio (HR) = 1.19, 95% confidence interval (CI) : 1.09–1.30], 90‐day entry into permanent care (HR = 1.96, 95% CI : 1.60–2.38), and increased likelihood of activities of daily living limitations (OR = 1.36, 95% CI  1.00–1.85).^25^ Other studies, such as Zerky et al, showed that dementia in very old medically ill inpatients was predictive of discharge to a nursing home in acute and rehabilitation geriatric hospitals.^26^

Health care outcomes for people living with dementia are dependent on the availability of appropriate services, which vary by region and country, being more likely to result in more inequitable outcomes for those with dementia when compared with outcomes for people without cognitive impairments.^27^ Although patients with dementia showed a lesser degree of FIM improvement than non‐dementia patients, the discharge FIM score still showed a significant improvement (mean motor FIM change – 17.6, *P* < 0.001). Consulting teams should carefully design goal‐orientated rehabilitation programs suitable for people with dementia to ensure optimum clinical outcomes following hip fracture. Dewing and Dijk's review stresses the importance of recognizing negative consequences that can be associated with inpatient and post discharge management of those with dementia due to health care personnel having insufficient understanding and the requirements associated with dementia.^28^ This study showed that there is an increased need for carer support and community support services (personal care, allied health care, meal preparation, provision of goods and equipment, transport, and case management) in the post‐rehabilitation phase, being greater for the dementia cohort.

### 6.1 Strengths and Limitations

This study is a large retrospective analysis of rehabilitation outcomes following hip fracture which examines the impact of underlying dementia in determining outcome by comparison with a matched non‐dementia cohort. Use of AROC data allowed for analysis of an extremely large data set as it incorporated all public hospital admissions within Australia, the period of analysis extending over 5 years (2014–2019).

A limitation of the study was the inability to delineate the severity of dementia and therefore its impact on rehabilitation outcomes. Non‐dementia group could have undiagnosed cognitive impairment but did not impact on rehabilitation at the time of admission and therefore not recorded. Although FIM scoring has both motor and cognitive components, FIM scoring is heavily weighted for motor functioning.

## 7 CONCLUSION

With the rising incidence of dementia and hip fractures associated with a globally aging population, providing optimal inpatient rehabilitation and post‐discharge support services for this vulnerable group is essential to maximize functional gains and reduce long‐term health costs. Despite the increased discharge to an RACF for the dementia group, we demonstrated that improvements in both motor and cognitive function are likely to have a beneficial effect on long‐term management. By identifying functional and cognitive limitations and assistance prior to admission, strategic points of intervention across the trajectory of recovery can be introduced thereby improving clinical outcomes. Future research is needed to delineate the impact of severity of dementia on rehabilitation outcome as the disease progresses.

## AUTHOR CONTRIBUTIONS

Y.L. was responsible for initial drafting and revising the article. J.W. contributed to idea generation and revision of the article. T.A. was responsible for data analysis and revision of article.

## FUNDING INFORMATION

The authors received no financial support for the research, authorship, and/or publication of this article.

## CONFLICT OF INTEREST STATEMENT

The authors declared no potential conflict of interest.

## ETHICS APPROVAL

Ethics approval for this study (NRR_ AROC_ 2020_04) was granted according to the AROC ethics approval from the University of Wollongong Human Research Ethics Committee (AROC HREC approval Number: 2019/ETH13154).

## INFORMED CONSENT

Waiver of consent was obtained from the Australasian Rehabilitation Outcomes Centre (AROC).
